# Treatment outcome and readmission risk among women in women-only versus mixed-gender drug treatment programs in Chile

**DOI:** 10.1016/j.jsat.2021.108616

**Published:** 2021-09-01

**Authors:** Carla F. Olivari, Andrés Gonzáles-Santa Cruz, Pia M. Mauro, Silvia S. Martins, Jaime Sapag, Jorge Gaete, Magdalena Cerdá, Alvaro Castillo-Carniglia

**Affiliations:** a Society and Health Research Center, Facultad de Humanidades, Universidad Mayor, Badajoz 130, Suite 1305, Las Condes, Santiago, Chile; b Department of Epidemiology, Columbia University Mailman School of Public Health, 722 West 168th St., New York, NY 10032, United States; c Department of Public Health and Family Medicine, Pontificia Universidad Católica de Chile, Diagonal Paraguay 362, Santiago, Chile; d Faculty of Education, Universidad de los Andes, Monseñor Alvaro del Portillo 12455, Las Condes, Santiago, Chile; e Research Center for School Mental Health, Faculty of Education (ISME), Universidad de los Andes, Chile; f Millennium Nucleus to Improve the Mental Health of Adolescents and Youths, Imhay, Santiago, Chile; g Department of Population Health and Center for Opioid Epidemiology and Policy, New York University Grossman School of Medicine, 180 Madison Avenue, New York, NY 10016, United States; h School of Public Health, Universidad Mayor, José Toribio Medina 38, Santiago, Chile

**Keywords:** Substance use disorder, Treatment, Gender, Chile

## Abstract

**Introduction:**

Traditional treatment programs for substance use disorder (SUD) tend to be male-dominated environments, which can negatively affect women’s access to treatment and related outcomes. Women’s specific treatment needs have led some providers to develop women-only SUD treatment programs in several countries. In Chile, women-only programs were only fully implemented in 2010. We compared treatment outcomes and readmission risk for adult women admitted to state-funded women-only versus mixed-gender SUD treatment programs in Chile.

**Methods:**

We used a registry-based retrospective cohort design of adult women in women-only (*N* = 8200) and mixed-gender (*N* = 13,178) SUD treatment programs from 2010 to 2019. The study obtained data from the National Drug and Alcohol Service from Chile. We used a multistate model to estimate the probabilities of experiencing treatment completion, discharge without completion (i.e., patient-initiated discharge and administrative discharge), or readmission, as well as the likelihood of being readmitted, conditioned on prior treatment outcome. We adjusted models for multiple baseline characteristics (e.g., substance use, socioeconomic).

**Results:**

Overall, 24% of women completed treatment and 54% dropped out of treatment. The proportion of patient-initiated discharges within the first three month was larger in women-only than in mixed-gender programs (19% vs. 12%). In both programs, women who completed treatment were more likely to experience readmission at three months, and one and three years. In the long term, women in the women-only programs were more likely to complete treatment than women in mixed-gender programs (34% vs. 23%, respectively). The readmission probability was higher among women who previously completed treatment than those who had a discharge without completion (40% vs 21% among women in women-only programs; 38% vs. 19% among women in mixed-gender programs, respectively); no differences occurred in the risk of readmission between women-only and mixed-gender programs.

**Conclusions:**

In terms of treatment outcomes and readmission risk, women-only programs had similar results to mixed-gender programs in Chile. The added value of these specialized programs should be addressed in further research.

## 1. Introduction

In residential or outpatient settings, substance use disorder (SUD) treatment interventions usually occur in mixed-gender group format (U. S. Department of Health and Human Services & General, 2016), where group interventions can be a powerful therapeutic tool for addressing SUDs (National Institute on Drug Abuse, 2020). If well guided, group treatment can direct groups to foster healthy attachments, provide positive peer reinforcement, strengthen self-expression, and help individuals to develop new social skills (U.S. Department of Health and Human Services & General, 2016). However, research has criticized the group approach as insensitive to gender-related dynamics (Greenfield et al., 2010). Evidence shows that while women with SUD seek treatment in mental health services, men are more likely to access specialized SUD treatment programs. This divide can lead to a male-dominated therapeutic environment, as males can be overrepresented in these settings (McHugh et al., 2018; Tuchman, 2010). In this context, interactional styles in group interventions may better suit hierarchical and confrontational “male cultural norms” (McHugh et al., 2018).

Overall, women are less likely than men to use illicit drugs and tend to initiate substance use later in life than men, but women experience a faster progression from substance use to dependence or abuse, and consequently a faster progression to first treatment entry. This accelerated progression to SUDs is usually associated with more severe SUD-related problems (Ait-Daoud et al., 2019; Agabio et al., 2016). Over the last three decades, though, the increased recognition of sex and gender differences in SUDs has led to the implementation of women-only treatment programs in many countries (Schleifer & Pol, 2017).

In Chile, women-only programs were fully established at a national level in 2010 under the technical support and funding of the National Service for Prevention and Rehabilitation of Drug and Alcohol Consumption of Chile (Servicio Nacional para la Prevención y Rehabilitación del Consumo de Drogas y Alcohol y Ministerio de Salud de Chile, 2018). This program was designed to improve treatment access and address the vulnerabilities that Chilean women with SUD face.

From a risk environment perspective (Collins et al., 2019; Rhodes, 2009), social vulnerability of women with SUD experience social vulnerabilities as a result of a range of macro- (economic, political, and social milieus) and micro-level environments (drug trafficking neighborhoods, intimate-partner violence, sex trade participation) that intersect with disadvantaged social locations to produce increased risk (Collins et al., 2019).

Data from Chilean SUD treatment programs depict the disadvantaged position of women with SUD, expressed in their lack of economic autonomy (i.e., income generation), housing instability, food insecurity, trauma/violence exposure, and stigmatization due to difficulties in accomplishing Latin American traditional gender roles, in which women are expected to be affectionate, submissive, and a faithful spouse; mother and family caregiver, in contrast to the independent, polygamous, and dominant Chilean *macho* man (Cianelli et al., 2008; Hawkins et al., 2017). Accordingly, 45% of women admitted to public treatment programs reported being unemployed, and 29% reported unpaid work. Among women with co-occurring mental health problems, 70% reported being victims of interpersonal violence from their partners or other family members (Valencia-Recabarren, 2015). Results from an RDS study of people who use cocaine base paste in Chile may be indicative of how the structural environment and situated contexts in Chile affect women with SUD. For example, women from this study reported a total monthly income of USD$150 (USD$68 under the poverty line in Chile), which was 50% lower than the income that men reported (Instituto de Sociología de la Pontificia Universidad Católica de Chile, 2015). Women also reported more insecure housing conditions and lower levels of education (Instituto de Sociología de la Pontificia Universidad Católica de Chile, 2015).

Hence, the implementation of gender-specific services in Chile represents an effort to provide a therapeutic alternative that may better suit the needs of women with SUD.

The SUD treatment programs system implemented in Chile provides professional therapeutic interventions and services, including social support, psychotherapy (individual and group format), mental health counseling, and basic pharmacological support, among others (Servicio Nacional para la Prevención y Rehabilitación del Consumo de Drogas y Alcohol, & Ministerio de Salud de Chile, 2012). In contrast to the general population program, the women-only program also includes gender-sensitive services such as childcare facilities, peri/postnatal care (i.e., adequate infrastructure and trained staff), and transportation services. Based on a gender-relational perspective, this program seeks to provide a safe and empathic therapeutic environment to address gender-specific needs and develop social skills such as validation, empowerment, and empathy, which have been considered critical for attachment and recovery in groups of women (Covington & Surrey, 1997; Greenfield et al., 2013). In addition, women-only programs in Chile include coeducational services to enhance parental skills development and the acquisition of skills to generate income. Women-only programs also play an important role in coordinating with other social services (e.g., legal system, primary health care services) that are critical to the recovery and social integration of women with SUDs (Consejo Nacional para el Control de Estupefacientes, 2007; Servicio Nacional para la Prevención y Rehabilitación del Consumo de Drogas y Alcohol y Ministerio de Salud de Chile, 2018).

In several Latin American countries, a critical limitation for research and program management has been the lack of centralized data systems. However, in Chile, this difficulty has been overcome due to the implementation of a centralized data registry system, SISTRAT, since 2010 (Centro de Estudios Justicia y Sociedad, & Pontificia Universidad Católica de Chile, 2020). This data system registers all patients admitted to adult SUD treatment programs in centers affiliated with SENDA’s network, and contains relevant patient information (i.e., sociodemographics, health status, and substance use history). In this context, the implementation of women-only programs and SISTRAT data offers us a unique opportunity to analyze the outcomes of women-only SUD treatment options in a Latin American context.

Given the scarce evidence on treatment quality and outcomes of state-funded women-only treatments in Chile (Centro de Estudios Justicia y Sociedad, & Pontificia Universidad Católica de Chile, 2020; Valencia-Recabarren, 2015), this study aimed to compare treatment outcomes and readmission risk between adult women admitted to state-funded women-only treatment programs and those admitted to mixed-gender SUD treatment programs in Chile.

## 2. Methods

### 2.1. Study design

We conducted a retrospective analysis of 21,378 records in SISTRAT for women in residential and outpatient SUD treatments between 2010 and the third quarter of 2019. All patient identification information was encrypted using an MD5 (Message Digest 5) algorithm, which is widely used in security protocols to encrypt information, such as a personal identification number, into a 128-bits hash code (Jayawickrama, 2008). The selected data encompass admissions and discharges for SUD treatment. The eligibility criteria included women of 18+ years of age admitted at least once to a women-only or mixed-gender SUD treatment program funded by SENDA between 2010 and 2019.

The Ethics Committee of Universidad Mayor, Chile, reviewed and approved this study (No. 260/2019).

### 2.2. Measures

A treatment center professional interview all clients of state-funded SUD treatment programs in Chile upon admission. Through this interview, the center’s professionals gather information on the client’s sociodemographic characteristics, health status, substance use patterns, among other factors, which they then enter into the SISTRAT system. Clinical information stems from the appraisal of the treatment team in consultation with a psychiatrist or physician with mental health experience, if necessary. Centers must collect the data to proceed to payment.

Treatment outcomes considered in our study were registered at discharge, with two possible categories: treatment completion and discharge without completion. This study collapsed and treated as one treatment referrals to another SUD treatment (i.e., treatment transfers) within the same treatment network (often suggested by the professional team of the center due to change of address, change of the treatment plan, or other justified reason) with fewer than 45 days of difference. These can be conceived as consecutive episodes provided by one or more providers. Referrals outside of SENDA’s network (e.g., mental and other health centers) that did not experience a readmission within the study period were treated as right censored, because the follow-up period ended before any of the treatment outcomes occurred (Jepsen et al., 2015).

SENDA’s guidelines define treatment completion as a discharge after achieving the goals determined in the patient’s individual treatment plan developed by the therapeutic team at admission. We grouped patient-initiated discharges (i.e., drop-outs) and administrative discharges (i.e., discharges due to serious misconduct against treatment norms) into one category called “discharge without completion” (Guerrero et al., 2013).

The study defined readmissions as having a second entry to a treatment program within SENDA’s network (Moon & Lee, 2020). If more than one readmission was recorded, we considered only the first in the multistate analysis. We based this decision on the fact that among readmitted women, 72% registered only one admission.

We compared patient-level sociodemographic, substance use, health, and treatment characteristics of women in mixed-gender and women-only programs (i.e., age, educational attainment, have children, housing, biopsychosocial status, primary substance at admission, frequency of use of primary substance, co-occurring SUD, treatment duration, and treatment modality). See Supplemental Table S1 for a detailed description of these variables.

### 2.3. Analysis

After applying exclusion criteria (<18 years of age and men) and data cleaning processes, missing data ranged from 0% to ~4.4% across the covariates. We implemented multivariate imputation by chained equations using the Amelia package (Honaker et al., 2011; Zhang, 2016), assuming data were missing at random (see the Supplemental material for additional details).

Since we studied concatenated events (admission-discharge-readmission), we implemented a multistate approach to incorporate all data in a single modeling process. Multistate models are an extension of competing-risk regression models that allow us to analyze event history data (Castañeda & Gerritse, 2010; Kulkarni et al., 2017). We specified a model of four states, starting with the initial admission as the first state, followed by treatment completion and discharge without completion as intermediate competing states, ending with readmission as the sole absorbing state (see the Supplemental material for additional information). The study assessed the proportional hazards assumption in the Cox models visually and through a chi-squared goodness-of-fit test (more details in the Supplemental material). We chose the parametric model that best fit the data for each transition according to Akaike information criterion (Akaike, 1976), visual inspection, and extrapolation up to 15 years. We compared intercept-only models to select the standard parametric models across each of the transitions. The best-fitted models included Gompertz distributions for transitions from admission to treatment completion and admission to discharge without completion. The study team chose log-normal distribution for the transition from admission to readmission. Generalized-Gamma and log-normal distributions, respectively, provided the most reasonable fit for the transitions from treatment completion and discharge without completion. Additional information on the modeling process is available in the Supplemental material.

We checked whether the time spent in a previous state played an important role for intermediate states. As treatment duration (i.e., time in the baseline state) could affect the likelihood of readmissions, we used a semi-Markov multistate model that included the time spent in treatment (years in treatment). This multistate model is the result of the combination of the selected distributions for each of the transitions. The study adjusted these models for all covariates listed in Section 2.2 and detailed in Table S1 in the Supplemental material. Based on two hypothetical patients, we first obtained the patient-specific instantaneous cumulative hazards of progressing from one state to another. From the adjusted models, we calculated transition probabilities (i.e., the probability that a patient would change from a state to another at a certain time), and length of stay in each state by simulating patients’ trajectories and transitions at three months, and 1 and 3 years, modeling at the individual patient level, which generated two hypothetical patients with the same average covariate characteristics, except for being exposed to different type of programs (Crowther & Lambert, 2017; Jackson, 2016; Williams et al., 2017). We considered these three time points based on a consensus established between our research team and SENDA’s team professionals who are responsible for treatment program design and functioning in Chile.

The study calculated the Kaplan-Meier survival curves and simple survival model using the *survival* package (Therneau T., 2021). The study estimated the smoothed hazard function through the *muhaz* package (Hess & Gentleman, 2019). The multistate semi-Markov models were fitted using the *mstate* (de Wreede et al., 2011)*, flexsurv* (Jackson, 2016), and *survival* packages. The research team completed all of the analyses in R statistical software version 4.0.2 (R Core Team, 2020).

Data and markdown with all software codes and outputs are available at https://bit.ly/34uGjbv.

## 3. Results

Table 1 summarizes demographic and substance use data comparing baseline characteristics of women attending women-only and mixed-gender treatment programs between 2010 and 2019. Samples differ from one another in several characteristics. The women-only programs had a larger proportion of women between 18 and 29 years old (41%) than the mixed-gender programs (34.3%); the proportion of women in women-only treatment who reported living in their own house was 19% lower than women in mixed-gender programs; and women in women-only programs were 1.28 times more likely to be staying temporarily with relatives than those in mixed-gender programs. A larger proportion of severe biopsychosocial status occurred in women in the women-only programs (57.4% vs. 30.6%). A greater proportion of women in women-only treatment declared using one or more drugs as secondary substances compared to patients in the mixed-gender program (79% vs. 66,7%). Finally, women in the women-only programs were 8.8 times more likely to be admitted to residential treatment settings than women in the mixed-gender programs.

The incidence rate of treatment readmission was approximately 85 per 1000 patients-year among women admitted to women-only treatment at baseline vs. 55 per 1000 patients-year among women admitted to mixed-gender programs. Additionally, the incidence rate of readmission among patients with a discharge without completion was approximately 83 vs. 63 per 1000 patients-year in patients with treatment completion at admission.

### 3.1. Multistate model

**Fig. 1.**
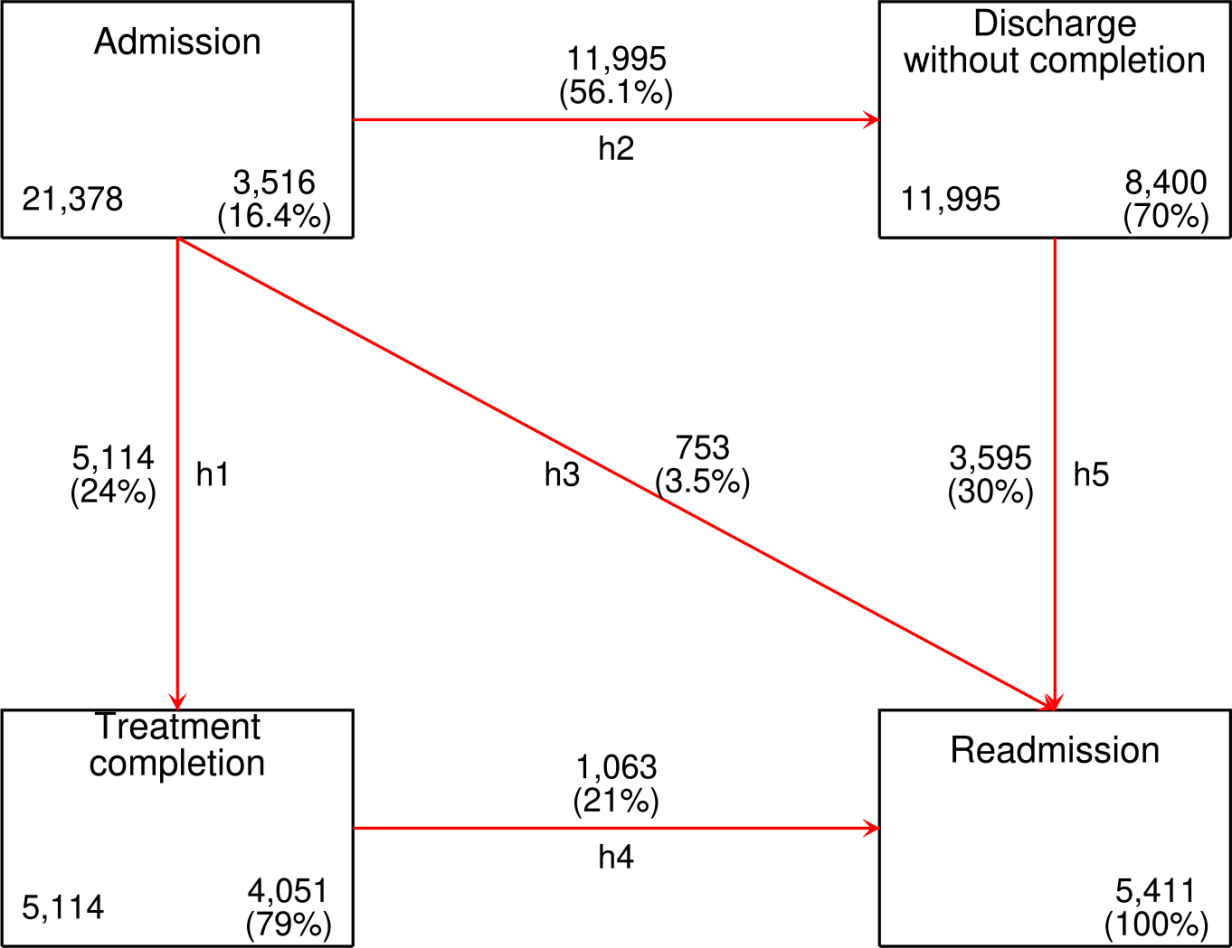
Graphical representation of four states model. Note. The number in the bottom-left corner depicts the number of cases that passed through the state; the number in the bottom-right corner shows no other events or subjects remaining in the same state; “h” stands for transitions. The 3516 users who remained in the Admission state had an ongoing treatment at baseline or were referred to other treatments but did not experience readmission.

As Fig. 1 shows, this model estimates the adjusted probability of 5 possible transitions. There were 21,378 women with at least one treatment admission during any time point between January 1, 2010, and November 13, 2019; 16.4% (*n* = 3516) remained in the same states up until the end of the follow-up period, mostly those admitted in 2018–2019, since they had a shorter follow-up period and thus a lower chance of completing their treatment; 56.1% had a discharge without completion; 24% completed treatment; and 3.5% transitioned directly from admission to readmission. The latter corresponds to women referred to treatments outside SENDA’s network who were then readmitted to treatment.

#### 3.1.1. Cumulative hazards

**Fig. 2.**
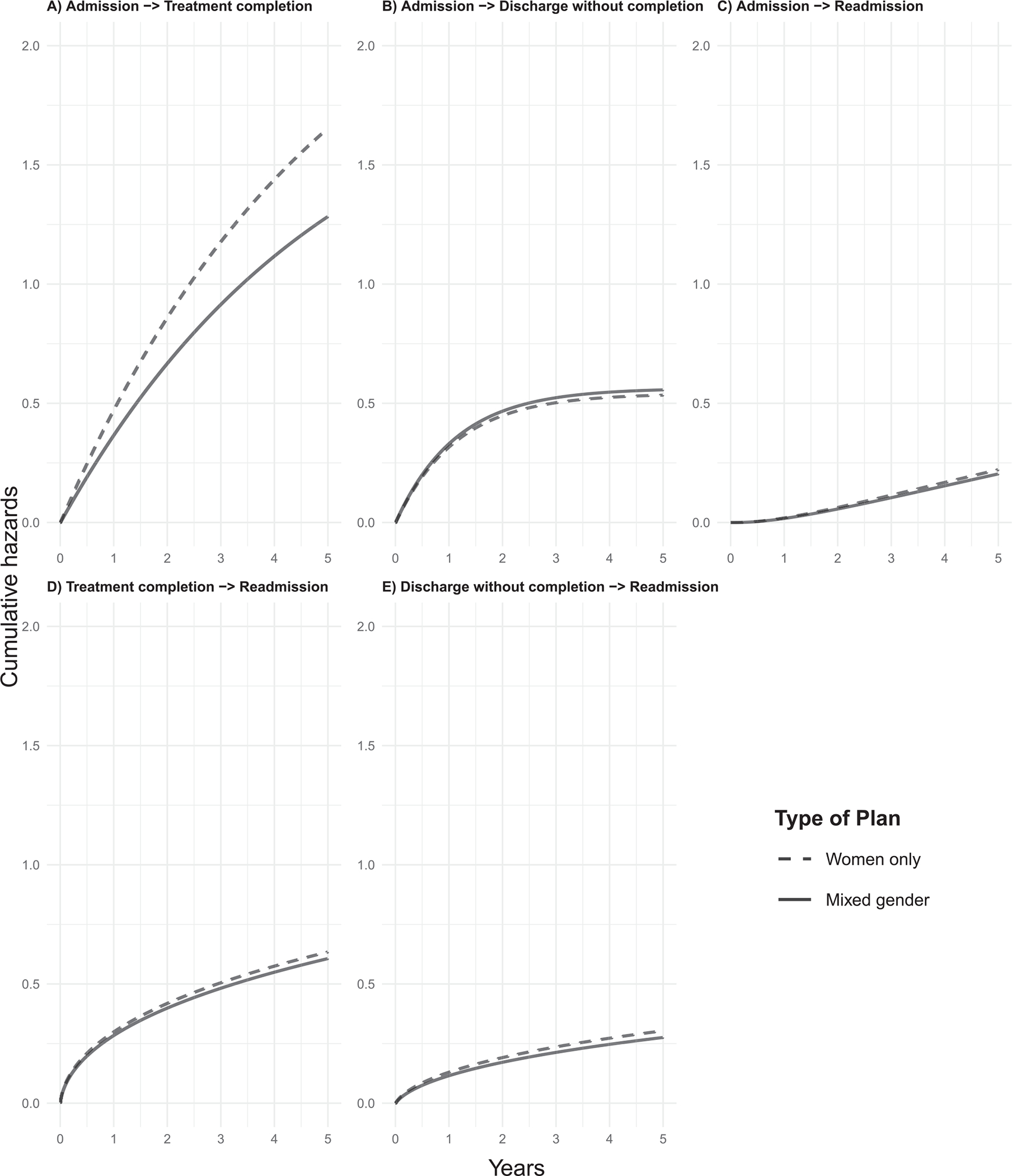
Cumulative hazards of transitioning across different states among women in women-only vs. mixed-gender treatment programs. Note. Dashed line: Women-only program. Solid line: Mixed-gender program. Both lines represent hazards for average covariate values, which are woman aged 30–39, who completed high school or less, reported alcohol as the primary substance at admission, had a daily consumption frequency, moderate biopsychosocial status (i.e., multidimensional clinical appraisal of SUD severity made by the professional team), stayed temporarily with a relative, had co-occurring SUD (i.e., diagnosis of abuse of dependence of one additional substance), had more than one child, and were in a residential treatment modality.

We computed the adjusted hazards of transitioning from one state to another based on a set of the most frequent categories of each covariate, also known as patient-specific transitions (Putter et al., 2007). As Fig. 2, panel A shows, women in women-only programs have higher adjusted hazards of treatment completion compared to women in mixed-gender programs. Women-only programs showed slightly lower rates from admission to discharge without completion. For the transitions admission-readmission, discharge without completion-readmission, and treatment completion-readmission, differences between women-only and mixed-gender were negligible, with slightly greater rates for women-only programs.

By incorporating these patient-specific cumulative hazards as input, we estimated the probability of transitioning from one state to another, and the predicted average time spent for the three follow-up periods considered (3 months, 1 year, and 3 years).

#### 3.1.2. Transition probabilities

Table 2 shows that the probability of experiencing readmission was significantly greater for those patients with treatment completion at every time point for both types of programs. The estimated state transition probabilities for the sets of covariates do not differ much between program types. However, we noticed that women-only programs were more likely to transition from admission to treatment completion than to discharge without completion. This difference is statistically significant only at 3 years (34%; 95% CI: 28–40% vs. 23%; 95% CI: 20–26%). In contrast, mixed-gender programs had a slightly greater transition probability from admission to discharge without completion. Still, these differences were not statistically significant at any time point that the study measured. The readmission probability was higher among women who previously experienced treatment completion than those who experienced a discharge without completion (40% vs 21% among women in women-only programs; 38% vs. 19% among women in mixed-gender programs, respectively); no differences existed in the probability of readmission between women-only and mixed-gender programs.

#### 3.1.3. Expected length of stay

The study found no significant differences between women-only and mixed-gender programs in the length they stayed in each state (e.g., average time between treatment discharge and readmission) at any time point reported (3 months, 1 year, and 3 years). However, for women-only programs, after one year of observation, those who were discharged without completion were expected to remain in that state on average for 0.92 (95% CI: 0.90–0.95) years vs. 0.82 (95% CI: 0.75–0.88) for those who experienced treatment completion. For women in mixed-gender programs, those who were discharged without completion remain in that state on average for 0.93 (95% CI: 0.91–0.95) years vs. 0.83 (95% CI: 0.75–0.89) for those who had treatment completion. The relative difference between those discharged without completion and those with a treatment completion is the same at 3 years (see the Supplemental material).

## 4. Discussion

Our study examined treatment outcomes for women in women-only versus mixed-gender SUD treatment programs using data of 21,378 adult women admitted to publicly funded SUD treatments from 2010 to 2019 in Chile. We found that patients admitted to women-only programs had a slightly higher probability of treatment completion than those admitted to mixed-gender programs. However, we observed no difference in the probability of being discharged without completion or in the probability of being readmitted to treatment. Overall, about one quarter of patients had completed treatment and a little less than half had a patient-initiated discharge (15% within the first 3 months). More women in women-only programs had a patient-initiated discharge within the first 3 months of treatment (19%) compared to women in mixed-gender programs (12%); the former were younger, reported using cocaine base paste daily in a larger proportion, and had a more severe biopsychosocial status.

Based on the available literature, the comparative effectiveness of women-only and mixed-gender programs remains unclear, with several studies showing mixed results. However, as expected under the rationale of gender-oriented programs, our results show that women in the women-only programs had a greater probability of completing treatment than those in the mixed-gender program, which research has been indicated as one of the most critical factors to obtaining treatment benefits overall (Brorson et al., 2013). This finding is also in line with previous evidence indicating that women in women-only programs had greater likelihood of staying longer in treatment relative to women in mixed-gender programs (Greenfield et al., 2007). In line with this, higher levels of treatment completion in programs have been associated with the availability of facilities that allow women to participate with their children, which research has identified as a critical barrier to treatment access for women (Brady & Ashley, 2005).

The negligible differences of readmission probabilities between programs are consistent with evidence showing no significant associations between the type of program (i.e., women-only or mixed-gender) and better long-term treatment outcomes (Hser et al., 2011; Kaskutas et al., 2005). For example, a randomized clinical trial that compared outcomes of women-only outpatient programs to mixed-gender programs (*N* = 122) found no significant differences in psychiatric or social problem severity at any of the follow-up points (6- and 12-months posttreatment). Similarly, the study found no significant differences in drug use abstinence between the two groups (Kaskutas et al., 2005). One of the unique contributions of this study is that it relies on a large population-based dataset. Additionally, to our knowledge, no previous studies on this topic have taken advantage of multistate modeling to look simultaneously at sequential events in SUD treatments. Our findings add valuable information on women-only treatments in the Latin American context, where little research has been published on women-only treatments’ comparative effectiveness (Zilberman, Tavares, et al., 2003).

Research has had an ongoing debate (Hansen et al., 2020; Hser et al., 2011) about readmission as a treatment outcome—its advantages and disadvantages, and its different interpretations. For example, Hansen et al. argue that readmission overcomes the difficulties of using measures based on self-reported data such as the definition of treatment goals and experiencing return to substance use (Hansen et al., 2020). Other authors have proposed nuances for interpreting readmission that can certainly apply to our results (Hornack & Yates, 2017; Hser et al., 2011). Readmission may be interpreted as a positive event if the readmission results from a woman’s increased awareness of her need to minimize drug exposure and skills development that has resulted in efficiently using health care services (Almeida et al., 2018; Vazquez et al., 2015). Women at risk of returning to substance use might have been able to seek counseling in primary care services as the first preventive action. If primary care support could not prevent return to use, then treatment readmission may have been needed as part of therapeutic planning. Thus, network coordination between primary care services and specialized services can maximize resources and ensure care continuity (Pan American Health Organization, 2011; Vazquez et al., 2009).

In contrast, if readmissions are the result of the existence of a “captive” population of specialized services, then readmission may be interpreted as a health system failure, one that has failed to offer care continuity in services of lower complexity (Pan American Health Organization, 2010), which are expected to be an important point of support for those with chronic illnesses such as SUDs.

In light of these interpretations, the negligible differences between women-only and mixed-gender programs do not allow us to assert that women admitted to women-only programs had a lower readmission risk after treatment completion than women in mixed-gender programs. Future studies should formally test differences in transition probabilities.

Our analytic approach allows us to capture only a part of a highly complex phenomenon that is influenced by several factors and that has multiple relevant outcomes apart from type of discharge and treatment readmission (i.e., social functioning, criminal offending, return to substance use, among others) that we could not consider in the analysis. Other intersecting dimensions, such as experiences of gender-based violence and economic dependence, may characterize risk environments that may lead to treatment readmission. For example, in Chile, women previously admitted to SUD treatment may prefer readmission because they lack the resources to maintain stable housing and food security. Especially in residential settings, individuals may see treatment readmission as a way to avoid intimate-partner violence, which research has shown to be highly prevalent among women with SUD. Beyond evidence of the effectiveness of women-only or mixed-gender programs, women-only programs are likely to be more sensitive to gender-specific needs (e.g., intimate partner violence, pregnancy) than mixed-gender programs and could enhance treatment access for women (Holzhauer et al., 2020).

### 4.1. Limitations

While our study used a large administrative dataset that had many advantages, the study also has limitations. For example, some relevant clinical information is not registered in SISTRAT, such as stage of change, traumatic experiences, or addiction severity. We acknowledge that the existence of these and other unmeasured variables may lead to residual confounding when comparing women-only and mixed-gender treatment programs. Other key variables that this study measured may vary in quality or completeness, such as psychiatric comorbidity. We did not have other information that could enhance our ability to describe complex trajectories of women outside SUD treatment programs. Data on mortality, hospitalizations, and incarceration could complement the analysis and point to potential milestones before readmission. Finally, due to the novel methodological framework that we used, we recognize that model structures influence conclusions (Cranmer et al., 2020), and that there is room for modeling improvements (e.g., more flexible transition probability distributions) and more complex structures, including other states in the treatment outcome (early vs. late dropout), second or third treatment readmissions, and time-varying confounders or interaction terms (Gran et al., 2015).

## 5. Conclusions

Overall, our findings suggest that women-only programs have similar results in terms of treatment outcomes and readmission risk to mixed-gender programs. Future research on the added value of specialized SUD treatment programs and their effectiveness should incorporate other dimensions of analysis to better capture the complexity of this phenomenon. For example, information on trauma histories, economic dependence, gender-based violence experiences, social support networks, and other services utilization (i.e., primary care services and hospitalizations) are relevant factors that may influence treatment success and readmission risk. Women’s preferences regarding SUD treatment and how the construct of “gender” held within treatment centers may affect women’s recovery process have been pointed out as critical dimensions that remain necessary to address (Grella, 2018; Neale et al., 2018). Qualitative research may be a better methodological approach to address these and other dimensions.

A multistate framework may be an adequate analytic approximation to jointly capture SUD treatment utilization as well as other health services utilization that the integrated health care network implemented in Chile has provided.

## Supplementary Material

Supplemental material

## Figures and Tables

**Table 1:** Characteristics of women admitted to women-only and mixed-gender treatment programs, Chile 2010–2019.

	Women-only (*N* = 8200)	Mixed-gender (N = 13,178)	Statistic	*p* value

Age in years at admission to treatment. N (%)			X^2^^a^(3)= 185.38	<0.001
18–29	3364 (41.0)	4522 (34.3)		
30–39	2754 (33.6)	4277 (32.5)		
40–49	1382 (16.9)	2651 (20.1)		
50+	700 (8.5)	1728 (13.1)		
Educational attainment. N (%)			X^2^^a^ (2) = 7.55	0.023
Completed primary school or less	2655 (32.4)	4360 (33.1)		
Completed high school or less	4281 (52.2)	6645 (50.4)		
More than high school	1264 (15.4)	2173 (16.5)		
Primary substance at admission. N (%)			X^2^^a^ (4) = 722.05	<0.001
Alcohol	1903 (23.2)	4843 (36.8)		
Cocaine hydrochloride	1435 (17.5)	2526 (19.2)		
Cocaine base paste	4116 (50.2)	4304 (32.7)		
Marijuana	474 (5.8)	943 (7.2)		
Other	272 (3.3)	562 (4.3)		
Consumption frequency of primary substance. N (%)			X^2^^a^ (4) = 814.09	<0.001
Less than 1 day per week	218 (2.7)	842 (6.4)		
1 day per week	295 (3.6)	1045 (7.9)		
2 to 3 days per week	1680 (20.5)	3891 (29.5)		
4 to 6 days per week	1175 (14.3)	2052 (15.6)		
Daily	4832 (58.9)	5348 (40.6)		
Biopsychosocial status. N (%)			X^2^^a^ (2) = 1703.06	<0.001
Mild	163 (2.0)	1329 (10.1)		
Moderate	3334 (40.7)	7823 (59.4)		
Severe	4703 (57.4)	4026 (30.6)		
Tenure status of households. N (%)			X^2^^a^ (4) = 258.47	<0.001
Illegal settlement	146 (1.8)	180 (1.4)		
Owner/transferred dwellings/pays dividends	2498 (30.5)	4936 (37.5)		
Renting	1355 (16.5)	2725 (20.7)		
Stays temporarily with a relative	3978 (48.5)	4994 (37.9)		
Others	223 (2.7)	343 (2.6)		
Co-occurring SUD. N (%)			X^2^^a^ (2) = 432.20	<0.001
No additional SUD	1727 (21.1)	4397 (33.4)		
One additional SUD	3215 (39.2)	4898 (37.2)		
More than one additional SUD	3258 (39.7)	3883 (29.5)		
Has children = Yes. N (%)	7287 (88.9)	11,522 (87.4)	X^2^^a^ (1) = 9.67	<0.001
Treatment outcome. N (%)			X^2^ (5) = 249.34	<0.001
Administrative discharge	744 (9.1)	994 (7.5)		
Early drop-out	1522 (18.6)	1638 (12.4)		
Late drop-out	2313 (28.2)	4784 (36.3)		
Ongoing treatment	573 (7.0)	1026 (7.8)		
Referral to another	1042 (12.7)	1628 (12.4)		
treatment				
Treatment completion	2006 (24.5)	3108 (23.6)		
Days in treatment. Mean (SD)	221.48 (190.78)	247.57 (198.18)	t^b^ = 9.58	<0.001
Treatment modality = Residential. N (%)	3323 (40.5)	603 (4.6)	X^2^^a^ (1) = 4354.71	<0.001

Note: Days in treatment with missing dates of discharge were calculated based on the difference between admission date and 2019-11-13.

a Chi-square test for independence.

b *t*-Statistic difference of means.

**Table 2:** Probability of remaining or transitioning to any of the four states, Chile 2010–2019.

Actual state	Women-only				Mixed-gender			
	ADM	TC	DWC	READM	ADM	TC	DWC	READM

At 3 months								
Admission	0.79 [0.77–0.81]	0.10 [0.09–0.12]	0.09 [0.08–0.10]	0.01 [0.01–0.02]	0.81 [0.79–0.83]	0.08 [0.07–0.10]	0.10 [0.09–0.11]	0.01 [0.01–0.02]
Treatment completion	-	0.86 [0.81–0.91]	-	0.14 [0.09–0.19]	-	0.87 [0.81–0.92]	-	0.13 [0.08–0.19]
Discharge without completion	-	-	0.95 [0.93–0.96]	0.05 [0.04–0.07]	-	-	0.95 [0.93–0.97]	0.05 [0.03–0.07]
At 1 year								
Admission	0.45 [0.40–0.48]	0.26 [0.22–0.30]	0.20 [0.18–0.23]	0.09 [0.07–0.12]	0.49 [0.45–0.53]	0.21 [0.18–0.25]	0.22 [0.20–0.25]	0.08 [0.06–0.10]
Treatment completion	-	0.74 [0.64–0.82]	-	0.26 [0.18–0.36]	-	0.75 [0.66–0.83]	-	0.25 [0.17–0.34]
Discharge without completion	-	-	0.88 [0.84–0.91]	0.12 [0.09–0.16]	-	-	0.89 [0.86–0.92]	0.11 [0.08–0.14]
At 3 years								
Admission	0.17 [0.13–0.20]	0.34 [0.28–0.40]	0.23 [0.20–0.26]	0.26 [0.21–0.32]	0.22 [0.17–0.26]	0.30 [0.25–0.36]	0.26 [0.23–0.29]	0.23 [0.18–0.28]
Treatment completion	-	0.60 [0.50–0.70]	-	0.40 [0.30–0.50]	-	0.62 [0.50–0.73]	-	0.38 [0.27–0.50]
Discharge without completion	-	-	0.79 [0.74–0.83]	0.21 [0.17–0.26]	-	-	0.81 [0.76–0.85]	0.19 [0.15–0.24]

Note. Removed Readmission rows because it was an absorbing state. ADM = Admission; TC = Treatment completion; DWC = Discharge without completion; READM = Readmission. Cells with “–” describe unallowed transitions.
