# Supplementary material for: Treatment outcome and readmission risk among women in women-only versus mixed-gender drug treatment programs in Chile
Source: J Subst Abuse Treat. Author manuscript; Available in PMC 2022 Apr 29. (PMC9052114; doi:10.1016/j.jsat.2021.108616)
Supplement: Supplemental material [file NIHMS1793634-supplement-Supplemental_material.docx]

# **Supplemental material**

**Treatment outcome and readmission risk among women in women-only versus mixed-gender drug treatment programs in Chile**

**Figure S1. Summary of Processes of Data Cleaning, Standardization & Imputation**



We obtained yearly treatment data from the Electronic Registration Systems for People in Treatment (SISTRAT) in Chile. Data were deidentified by encrypting the Chilean Unique National Role (RUN) into an MD5 hash algorithm. Posteriorly, some cases had to be removed because of duplication of records (e.g., same treatment registered in two or more annual datasets), overlapped treatments, or unrealistically long treatments (>1095 days). Additionally, we normalized some fundamental values of records such as date of admission, discharge, age at admission, and ambiguous time-invariant values because of inconsistencies in more than one record (e.g., at readmission) for each user (e.g., date of birth or sex). According to the conceptualization of referrals provided by SENDA professionals, individual admission episodes were allocated to the same treatment if fewer than 45 days elapsed between referrals and initiation of a subsequent treatment. These can be conceived as consecutive episodes provided by one or more providers. Once treatments were collapsed, missing values in the variables of interest were imputed, assuming values were missing at random. Tenure status of households was the variable with the highest number of missing values (4.4%, n= 942), followed by the biopsychosocial status (1.7%, n=370).

**Specifics of Multistate models**

The statistical modeling needs to consider both treatment outcomes and readmission. Multi-state modeling, an extension of competing-risk models, provides a framework that allows analyzing event history data (Castañeda & Gerritse, 2010; Kulkarni et al., 2017).

We used a continuous-time state-transition framework with no recurring states, starting from partitioning events into states of interest (Williams, Lewsey, Briggs, & Mackay, 2016). The rationale for model selection relied on researchers' knowledge and clinical guidance (Gibson et al., 2019).

The model considers the initial admission state, followed by two competing events whether a patient had a treatment completion or discharge without completion on their baseline treatment, and the fourth state is readmission, which in this case is an absorbing state.

All the analyses were based on the knowledge of fully observed and exact transition times (subject to right censoring in the survival cases). A matrix of plausible transitions was defined for both models (Bullement, Cranmer, & Shields, 2019; Gibson et al., 2019).

We estimated the event times as the difference between admission and the date of the event of interest, or the last follow-up (November 13, 2019) for censored cases. All individuals were censored in the following situations: referral to a treatment outside SENDA’s network and discharged or readmitted after the follow-up period. Simultaneous transitions were transformed by subtracting a day if the patient immediately transited to an intermediate state and adding one day to the absorbing event if the patient was immediately readmitted after finishing the baseline treatment.

We visually inspected proportionality among transitions as stated in Williams et al. (2016) of the proportional hazard assumption through graphically plotting log cumulative baseline hazards against log analysis time and log(−log(survival)) vs. log(time) plots for each transition to evaluate the appropriateness (Bradburn, Clark, Love, & Altman, 2003; Eulenburg, Mahner, Woelber, & Wegscheider, 2015).

Markov processes assume that the transition to a future state does not depend on a past state and time spent in it, but solely on the present state (Cao et al., 2015). We contrasted the Markov assumption with a state-arrival extended model, in which the time of arrival to an intermediate state is included as a covariate. Markov property should be relaxed if this covariate shows a significant coefficient, as in our case (Putter, Fiocco, & Geskus, 2007; Williams et al., 2016). Thus, we fitted survival models for each transition and included the time of arrival at the state as a covariate, comparing several transition-specific distributions (Crowther & Lambert, 2017). We compared nine standard survival parametric models with different distributions, including the exponential (assumes a constant hazard), weibull and gompertz (assume a monotonically increasing or decreasing hazard), the log-logistic and lognormal (allow for non-monotonic hazards), and more flexible distributions such as generalized F, gamma and generalized gamma (see Figure S3) (Bradburn et al., 2003; Dessie, Zewotir, Mwambi, & North, 2020; Gray et al., 2020; Jackson, 2016; Kearns, Stevens, Ren, & Brennan, 2020).

The resultant models were chosen based on a comprehensive combination of visual assessment of the goodness of fit of the predicted versus observed hazards (Hess & Gentleman, 2019), lower Akaike Information Criterion on transitions (in a multistate framework analysts may not rely solely on this criterion), and reasonable or plausible long-term extrapolations up 15 years since entry to a state (see Figure S3,Table S2 and Table S3) (Bensimon et al., 2020; Hoff et al., 2018; Smare, Lakhdari, Doan, Posnett, & Johal, 2020; Williams et al., 2016).

We calculated the transition intensities and cumulative hazards to obtain predicted probabilities, known as the instantaneous probability of transition from one state to another. Since it may be impractical or infeasible to do such calculations for the combination of every set of covariates (Hoff et al., 2018), we used a stratified approach by estimating the transition intensities for one individual conditioned on relevant covariate information: woman aged between 30 and 39 that completed high school or less, alcohol user, daily user, moderate biopsychosocial status, stay temporarily with a relative, using one additional substance has children, and attended a residential program. The only factor that differed between the two hypothetical women was being admitted to a women-only or mixed-gender treatment program (Gran, Lie, Øyeflaten, Borgan, & Aalen, 2015).

Posteriorly, and based on patient-specific transition intensities, we calculated the transition probabilities, or the probability that the process is in a given state at a fixed time, considering the current state and conditionally on the past trajectory until this fixed time. Considering that we were using sojourn times (i.e., time spent in each state), we adopted a generalized simulation algorithm to obtain transition probabilities and other quantities of interest (Crowther & Lambert, 2017; Jackson, 2016). The probability matrix was obtained by simulating the default 100,000 individual trajectories (de Wreede, Fiocco, & Putter, 2011; Jackson, 2016; Titman, 2015). We additionally repeated the estimation 1,000 times by default from the asymptotic normal distribution to obtain the corresponding confidence intervals (Jackson, 2016).

Finally, we calculated the expected total lengths of time in each state, or the amount of time spent in a determined state, conditioned on the starting state, at three different time windows: three months, one, and three years. This measure is equivalent to a restricted mean survival time for multistate models, which has also been considered an alternative for hazards (Rulli et al., 2018; Touraine, Helmer, & Joly, 2013). We compared the expected duration in each state between the women-specific and mixed-gender programs (Pedersen & Bjorner, 2017).

**Table S1. Patient characteristics used as covariates**

| **Sociodemographic** |  |
| --- | --- |
| Age category at admission | 18-29, 30-39, 40-49, and 50 or more. |
| Educational attainment | Defined as the last or the highest educational level completed by the patient. Categories were “Completed primary school or lower”, “Completed high school or lower”, and “More than high school”. |
| Have children | Yes/no. |
| Housing at the time of admission | which considers the categories “Stays temporarily with a relative”, “Renting”, “Illegal settlement”, “Owner/Transferred dwellings/Pays mortgage”, and “Others”, grouping other housing conditions not included in the previous categories. |
| **Substance use, health and treatment characteristics** |  |
| Biopsychosocial status | A clinical appraisal from professionals in the treatment team, which considers the level of withdrawal symptoms, the motive of admission, motivation to change, the severity of SUD, number of previous treatments, number of physical complaints, and characteristics of the social environment (e.g., family functioning). Is coded as mild, moderate, and severe (Vega-González & Pérez, 2021). |
| Primary substance at the first admission | Recorded by the therapeutic team and defined as the substance recognized by the patients as causing more problems at admission. |
| Frequency of use of primary substance | Days of use in the last 30 days previous to treatment. Categories considered “Less than 1 day per week”, “1 day per week”, “2-3 days per week”, “4-6 days per week”, and “Daily”. Those reporting no use in the last 30 days (1.80%) were grouped with those reporting less than one day a week (3.16%). |
| Co-occurring SUD | Coded as “No other SUD”, “One additional SUD”, and “More than one additional SUD”. |
| Treatment duration | Reported as the mean number of days in treatment. |
| Treatment modality | Report the proportion of patients in residential treatments vs. outpatient programs. |

**Figure S2. Log-log & cumulative hazard plots for each transition**

**
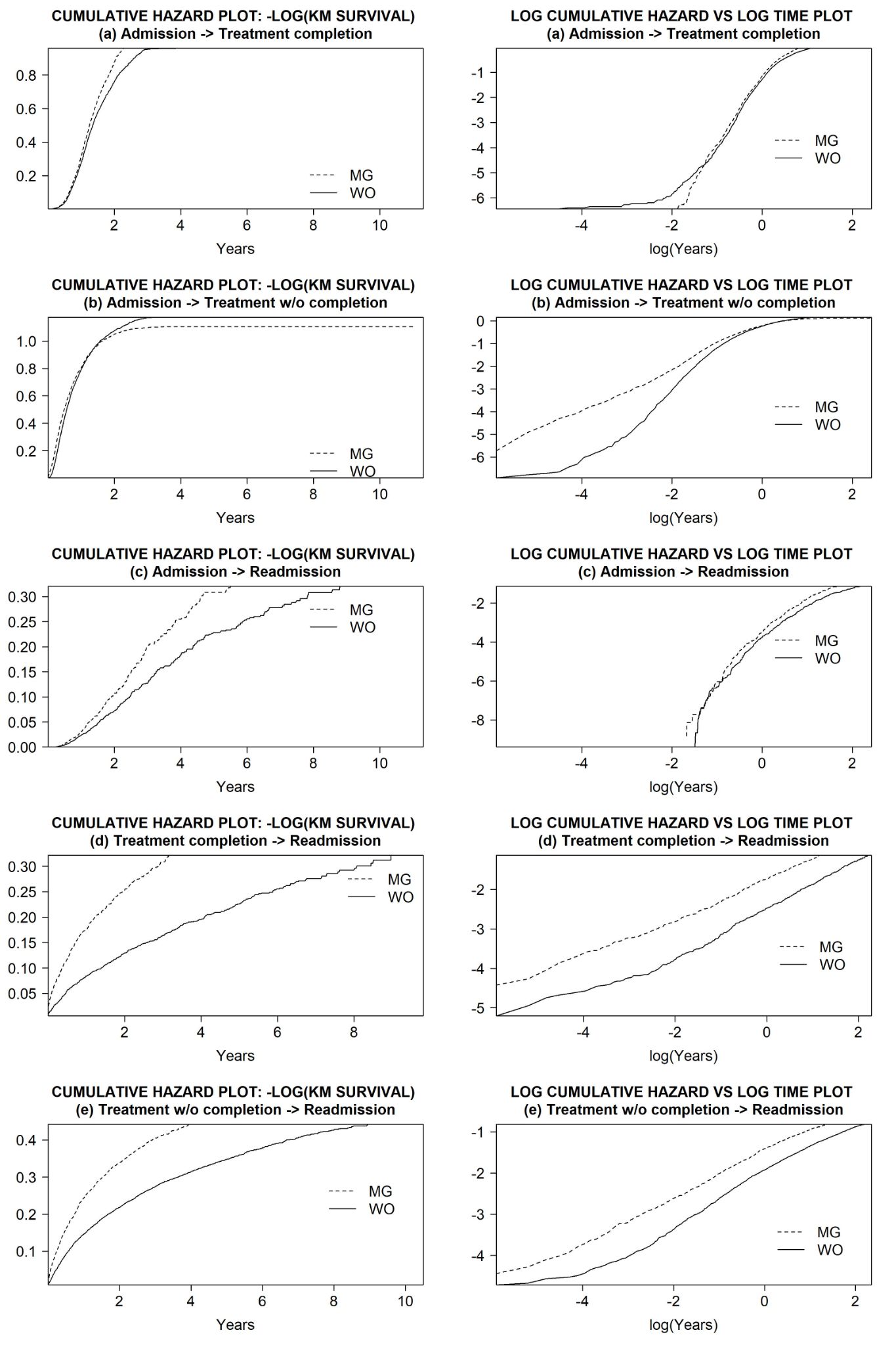
**

*Note. MG= Mixed-gender programs; WO= Women-only programs.*

As seen in Figure S2, we did not see parallel trends in every transition, and the test indicated a significant deviation from expected proportionality in Admission to Treatment without completion (X²(df=1)=226.67, p<0.001), Treatment completion to Readmission (X²(df=1)=10.34, p=0.001), and Treatment without completion to Readmission (X²(df=1)=34.26, p<0.001). Hence, we decided to select standard parametric distributions for each transition that do not solely rely on this proportionality assumption (Williams et al., 2016).

**Figure S3. Predicted hazard curves for the five transitions of the four-states multistate model.**


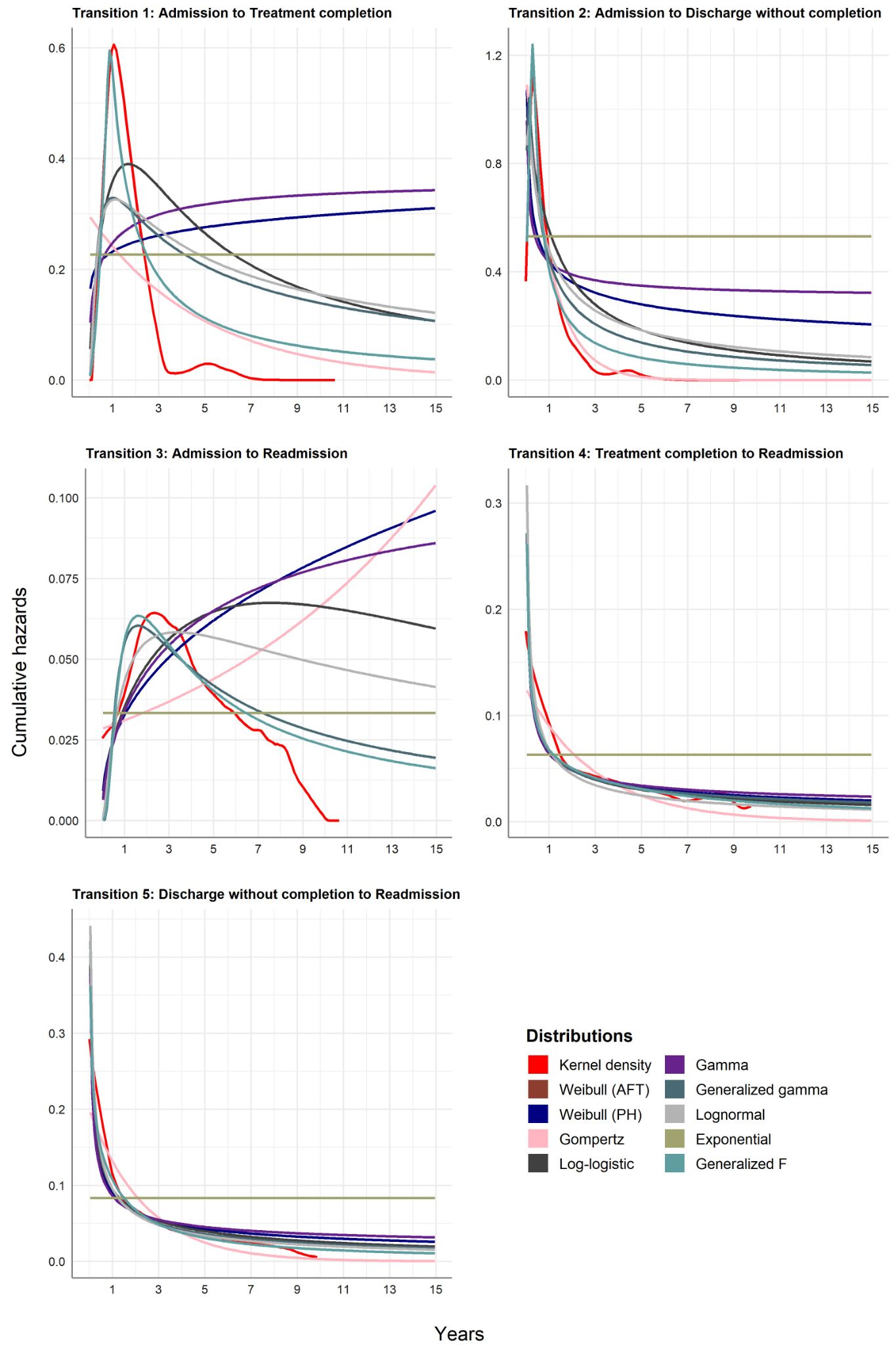


Note. Intercept-only models

**Table S2. Akaike Information Criterion for multistate models with different transition probability distribution**

| **Intercept-only models** | | | **Controlling for covariates** | | |
| --- | --- | --- | --- | --- | --- |
| **Distribution** | **Transition** | **AIC** | **Distribution** | **Transition** | **AIC** |
| Generalized F | 1 | 20,918 | Generalized F | 1 | 20,692 |
| Generalized gamma | 1 | 23,268 | Generalized gamma | 1 | 22,974 |
| Lognormal | 1 | 23,295 | Lognormal | 1 | 23,014 |
| Log-logistic | 1 | 23,308 | Log-logistic | 1 | 23,014 |
| Gompertz | 1 | 24,884 | Gompertz | 1 | 24,553 |
| Gamma | 1 | 25,017 | Gamma | 1 | 24,581 |
| Weibull (AFT) | 1 | 25,299 | Weibull (AFT) | 1 | 24,866 |
| Weibull (PH) | 1 | 25,299 | Weibull (PH) | 1 | 24,866 |
| Exponential | 1 | 25,419 | Exponential | 1 | 25,023 |
| Generalized F | 2 | 31,038 | Generalized F | 2 | 29,803 |
| Gompertz | 2 | 31,787 | Gompertz | 2 | 30,612 |
| Generalized gamma | 2 | 33,136 | Generalized gamma | 2 | 31,570 |
| Lognormal | 2 | 33,490 | Log-logistic | 2 | 32,097 |
| Log-logistic | 2 | 33,521 | Lognormal | 2 | 32,117 |
| Weibull (AFT) | 2 | 36,550 | Weibull (AFT) | 2 | 35,311 |
| Weibull (PH) | 2 | 36,550 | Weibull (PH) | 2 | 35,311 |
| Gamma | 2 | 37,582 | Gamma | 2 | 36,328 |
| Exponential | 2 | 39,166 | Exponential | 2 | 37,657 |
| Generalized F | 3 | 6,182 | Generalized gamma* | 3 | 6,132 |
| Generalized gamma | 3 | 6,182 | Lognormal | 3 | 6,175 |
| Lognormal | 3 | 6,242 | Log-logistic | 3 | 6,305 |
| Log-logistic | 3 | 6,373 | Gamma | 3 | 6,326 |
| Gamma | 3 | 6,389 | Weibull (AFT) | 3 | 6,365 |
| Weibull (AFT) | 3 | 6,424 | Weibull (PH) | 3 | 6,365 |
| Weibull (PH) | 3 | 6,424 | Gompertz | 3 | 6,548 |
| Gompertz | 3 | 6,603 | Exponential | 3 | 6,574 |
| Exponential | 3 | 6,629 | Generalized F*** | 3 | 8,379 |
| Log-logistic | 4 | 7,438 | Generalized F | 4 | 7,089 |
| Weibull (AFT) | 4 | 7,439 | Log-logistic | 4 | 7,090 |
| Weibull (PH) | 4 | 7,439 | Generalized gamma | 4 | 7,092 |
| Generalized gamma | 4 | 7,440 | Lognormal | 4 | 7,100 |
| Generalized F | 4 | 7,441 | Weibull (AFT) | 4 | 7,113 |
| Gamma | 4 | 7,442 | Weibull (PH) | 4 | 7,113 |
| Lognormal | 4 | 7,467 | Gamma | 4 | 7,136 |
| Gompertz | 4 | 7,672 | Gompertz | 4 | 7,330 |
| Exponential | 4 | 8,004 | Exponential | 4 | 7,692 |
| Generalized F | 5 | 22,799 | Generalized F | 5 | 22,335 |
| Generalized gamma | 5 | 22,832 | Generalized gamma | 5 | 22,366 |
| Lognormal | 5 | 22,841 | Lognormal | 5 | 22,371 |
| Log-logistic | 5 | 22,857 | Log-logistic | 5 | 22,397 |
| Weibull (AFT) | 5 | 22,928 | Weibull (AFT) | 5 | 22,503 |
| Weibull (PH) | 5 | 22,928 | Weibull (PH) | 5 | 22,503 |
| Gamma | 5 | 22,997 | Gamma | 5 | 22,600 |
| Gompertz | 5 | 23,296 | Gompertz | 5 | 22,853 |
| Exponential | 5 | 25,056 | Exponential | 5 | 24,602 |

Note. Transition numbers: 1= Admission to Treatment completion; 2= Admission to Discharge without completion; 3= Admission to Readmission; 4= Treatment completion to Readmission; 5= Discharge without completion to Readmission.

* Due to convergence issues, we defined the third transition for the Generalized gamma distribution using initial values of 0.0001 and 1.056 (mean of the time in the transition). The generalized F also presented convergence issues that made infeasible an expansion of the upper and lower confidence bounds.

**Table S3. Root Mean Square Error of Distributions and Transitions by Type of Program**

| Distribution | Transition | Mixed-gender | Women-only | Mean |
| --- | --- | --- | --- | --- |
| Generalized F | 1 | 0,073 | 0,081 | 0,077 |
| **Gompertz** | **1** | **0,118** | **0,088** | **0,103** |
| Generalized gamma | 1 | 0,156 | 0,140 | 0,148 |
| Lognormal | 1 | 0,172 | 0,161 | 0,167 |
| Log-logistic | 1 | 0,197 | 0,186 | 0,191 |
| Exponential | 1 | 0,203 | 0,243 | 0,223 |
| Weibull (AFT) | 1 | 0,249 | 0,328 | 0,288 |
| Weibull (PH) | 1 | 0,249 | 0,328 | 0,288 |
| Gamma | 1 | 0,274 | 0,355 | 0,315 |
| Generalized F | 2 | 0,068 | 0,086 | 0,077 |
| **Gompertz** | **2** | **0,070** | **0,087** | **0,078** |
| Generalized gamma | 2 | 0,113 | 0,132 | 0,123 |
| Lognormal | 2 | 0,151 | 0,171 | 0,161 |
| Log-logistic | 2 | 0,160 | 0,182 | 0,171 |
| Weibull (AFT) | 2 | 0,195 | 0,214 | 0,205 |
| Weibull (PH) | 2 | 0,195 | 0,214 | 0,205 |
| Gamma | 2 | 0,231 | 0,257 | 0,244 |
| Exponential | 2 | 0,339 | 0,383 | 0,361 |
| Generalized gamma | 3 | 0,015 | 0,021 | 0,018 |
| **Lognormal** | **3** | **0,018** | **0,022** | **0,020** |
| Log-logistic | 3 | 0,022 | 0,027 | 0,024 |
| Exponential | 3 | 0,020 | 0,029 | 0,025 |
| Weibull (AFT) | 3 | 0,022 | 0,028 | 0,025 |
| Weibull (PH) | 3 | 0,022 | 0,028 | 0,025 |
| Gamma | 3 | 0,022 | 0,028 | 0,025 |
| Gompertz | 3 | 0,022 | 0,029 | 0,025 |
| Generalized F | 3 | 0,033 | 0,043 | 0,038 |
| Lognormal | 4 | 0,016 | 0,051 | 0,034 |
| **Generalized gamma** | **4** | **0,017** | **0,052** | **0,035** |
| Generalized F | 4 | 0,018 | 0,052 | 0,035 |
| Log-logistic | 4 | 0,018 | 0,052 | 0,035 |
| Gamma | 4 | 0,018 | 0,052 | 0,035 |
| Weibull (AFT) | 4 | 0,018 | 0,052 | 0,035 |
| Weibull (PH) | 4 | 0,018 | 0,052 | 0,035 |
| Gompertz | 4 | 0,019 | 0,053 | 0,036 |
| Exponential | 4 | 0,023 | 0,055 | 0,039 |
| **Lognormal** | **5** | **0,025** | **0,063** | **0,044** |
| Generalized gamma | 5 | 0,026 | 0,064 | 0,045 |
| Generalized F | 5 | 0,026 | 0,064 | 0,045 |
| Gompertz | 5 | 0,027 | 0,066 | 0,047 |
| Log-logistic | 5 | 0,028 | 0,066 | 0,047 |
| Weibull (AFT) | 5 | 0,028 | 0,066 | 0,047 |
| Weibull (PH) | 5 | 0,028 | 0,066 | 0,047 |
| Gamma | 5 | 0,028 | 0,067 | 0,048 |
| Exponential | 5 | 0,042 | 0,082 | 0,062 |

Note. Transition numbers: 1= Admission to Treatment completion; 2= Admission to Discharge without completion; 3= Admission to Readmission; 4= Treatment completion to Readmission; 5= Discharge without completion to Readmission.

We chose the Gompertz distribution for the first and second transition, because this distribution provided a more reasonable extrapolation at 11 years, resembling most to the hazard curve, as seen in Figure S3, despite the Gompertz distribution showed the fifth and second best fit, respectively (AIC= 24,884 and AIC= 31,787). Considering that Generalized F & Generalized Gamma distributions showed convergence issues in the model that controlled for covariates, we selected the Log-normal distribution (AIC= 6,242).

Visual differences may seem difficult to appreciate for the fourth and fifth transitions. However, and despite this distribution was fourth in the AIC (but with negligible differences between the first six distributions), we chose Generalized gamma (AIC= 7,440) for the fourth transition because of the lower mean RMSE of both programs (see Table S3), and lower observed differences with smoothed hazard function. For the fifth transition, we chose Log-normal distribution despite being the third with lower AICs (AIC= 22,841) because of lower mean RMSE of both programs and lower observed differences with smoothed hazard function.

**Table S4. Expected total length of stay in years (and 95% confidence intervals)**

| **State** | **Time** | **Women-only** | **Mixed-gender** |
| --- | --- | --- | --- |
| **Admission** |  |  |  |
|  | ~3 months | 0.22 [0.22-0.22] | 0.22 [0.22-0.23] |
|  | ~1 year | 0.67 [0.64-0.69] | 0.69 [0.67-0.72] |
|  | ~3 years | 1.21 [1.10-1.32] | 1.34 [1.22-1.44] |
| **Treatment completion** |  |  |  |
|  | ~3 months | 0.23 [0.22-0.24] | 0.23 [0.22-0.24] |
|  | ~1 year | 0.82 [0.75-0.88] | 0.83 [0.75-0.89] |
|  | ~3 years | 2.15 [1.87-2.40] | 2.18 [1.88-2.44] |
| **Discharge without completion** |  |  |  |
|  | ~3 months | 0.24 [0.24-0.24] | 0.24 [0.24-0.25] |
|  | ~1 year | 0.92 [0.90-0.95] | 0.93 [0.91-0.95] |
|  | ~3 years | 2.58 [2.46-2.68] | 2.62 [2.50-2.72] |

Note. Since Treatment completion and Discharge without completion were transient states, the lengths of stay were calculated starting from these states.

**References**

Bensimon, A. G., Zhou, Z.-Y., Jenkins, M., Song, Y., Gao, W., Signorovitch, J., . . . Aguiar-Ibáñez, R. (2020). An Economic Evaluation of Pembrolizumab Versus Other Adjuvant Treatment Strategies for Resected High-Risk Stage III Melanoma in the USA. *Clinical Drug Investigation, 40*(7), 629-643. doi:10.1007/s40261-020-00922-6

Bradburn, M. J., Clark, T. G., Love, S. B., & Altman, D. G. (2003). Survival Analysis Part III: Multivariate data analysis – choosing a model and assessing its adequacy and fit. *British Journal of Cancer, 89*(4), 605-611. doi:10.1038/sj.bjc.6601120

Bullement, A., Cranmer, H. L., & Shields, G. E. (2019). A Review of Recent Decision-Analytic Models Used to Evaluate the Economic Value of Cancer Treatments. *Applied Health Economics and Health Policy, 17*(6), 771-780. doi:10.1007/s40258-019-00513-3

Cao, Q., Buskens, E., Feenstra, T., Jaarsma, T., Hillege, H., & Postmus, D. (2015). Continuous-Time Semi-Markov Models in Health Economic Decision Making: An Illustrative Example in Heart Failure Disease Management. *Medical Decision Making, 36*(1), 59-71. doi:10.1177/0272989X15593080

Castañeda, J., & Gerritse, B. (2010). Appraisal of Several Methods to Model Time to Multiple Events per Subject: Modelling Time to Hospitalizations and Death. *Revista Colombiana de Estadística, 33*(1), 43-61. Retrieved from <https://www.redalyc.org/articulo.oa?id=89915370004>

Crowther, M. J., & Lambert, P. C. (2017). Parametric multistate survival models: Flexible modelling allowing transition-specific distributions with application to estimating clinically useful measures of effect differences. *Statistics in Medicine, 36*(29), 4719-4742. doi:<https://doi.org/10.1002/sim.7448>

de Wreede, L. C., Fiocco, M., & Putter, H. (2011). mstate: An R Package for the Analysis of Competing Risks and Multi-State Models. *Journal of Statistical Software; Vol 1, Issue 7 (2011)*. Retrieved from <https://www.jstatsoft.org/v038/i07>

Dessie, Z. G., Zewotir, T., Mwambi, H., & North, D. (2020). Modelling HIV disease process and progression in seroconversion among South Africa women: using transition-specific parametric multi-state model. *Theoretical Biology and Medical Modelling, 17*(1), 10. doi:10.1186/s12976-020-00128-5

Eulenburg, C., Mahner, S., Woelber, L., & Wegscheider, K. (2015). A systematic model specification procedure for an illness-death model without recovery. *PloS one, 10*(4), e0123489-e0123489. doi:10.1371/journal.pone.0123489

Gibson, E. J., Begum, N., Koblbauer, I., Dranitsaris, G., Liew, D., McEwan, P., . . . Pritchard, C. (2019). Cohort versus patient level simulation for the economic evaluation of single versus combination immuno-oncology therapies in metastatic melanoma. *Journal of Medical Economics, 22*(6), 531-544. doi:10.1080/13696998.2019.1569446

Gran, J. M., Lie, S. A., Øyeflaten, I., Borgan, Ø., & Aalen, O. O. (2015). Causal inference in multi-state models–sickness absence and work for 1145 participants after work rehabilitation. *BMC Public Health, 15*(1), 1082. doi:10.1186/s12889-015-2408-8

Gray, J., Sullivan, T., Latimer, N. R., Salter, A., Sorich, M. J., Ward, R. L., & Karnon, J. (2020). Extrapolation of Survival Curves Using Standard Parametric Models and Flexible Parametric Spline Models: Comparisons in Large Registry Cohorts with Advanced Cancer. *Medical Decision Making, 41*(2), 179-193. doi:10.1177/0272989X20978958

Hoff, R., Corbett, K., Mehlum, I. S., Mohn, F. A., Kristensen, P., Hanvold, T. N., & Gran, J. M. (2018). The impact of completing upper secondary education - a multi-state model for work, education and health in young men. *BMC Public Health, 18*(1), 556. doi:10.1186/s12889-018-5420-y

Jackson, C. H. (2016). flexsurv: A Platform for Parametric Survival Modeling in R. *Journal of Statistical Software, 70*, i08. doi:10.18637/jss.v070.i08

Kearns, B., Stevens, J., Ren, S., & Brennan, A. (2020). How Uncertain is the Survival Extrapolation? A Study of the Impact of Different Parametric Survival Models on Extrapolated Uncertainty About Hazard Functions, Lifetime Mean Survival and Cost Effectiveness. *PharmacoEconomics, 38*(2), 193-204. doi:10.1007/s40273-019-00853-x

Kulkarni, S., Hall, I., Formica, R., Thiessen, C., Stewart, D., Gan, G., . . . Deng, Y. (2017). Transition probabilities between changing sensitization levels, waitlist activity status and competing-risk kidney transplant outcomes using multi-state modeling. *PloS one, 12*(12), e0190277. doi:10.1371/journal.pone.0190277

Pedersen, J., & Bjorner, J. B. (2017). Worklife expectancy in a cohort of Danish employees aged 55–65 years - comparing a multi-state Cox proportional hazard approach with conventional multi-state life tables. *BMC Public Health, 17*(1), 879. doi:10.1186/s12889-017-4890-7

Putter, H., Fiocco, M., & Geskus, R. B. (2007). Tutorial in biostatistics: competing risks and multi-state models. *Statistics in Medicine, 26*(11), 2389-2430. doi:<https://doi.org/10.1002/sim.2712>

Rulli, E., Ghilotti, F., Biagioli, E., Porcu, L., Marabese, M., D’Incalci, M., . . . Torri, V. (2018). Assessment of proportional hazard assumption in aggregate data: a systematic review on statistical methodology in clinical trials using time-to-event endpoint. *British Journal of Cancer, 119*(12), 1456-1463. doi:10.1038/s41416-018-0302-8

Smare, C., Lakhdari, K., Doan, J., Posnett, J., & Johal, S. (2020). Evaluating Partitioned Survival and Markov Decision-Analytic Modeling Approaches for Use in Cost-Effectiveness Analysis: Estimating and Comparing Survival Outcomes. *PharmacoEconomics, 38*(1), 97-108. doi:10.1007/s40273-019-00845-x

Titman, A. C. (2015). Transition probability estimates for non-Markov multi-state models. *Biometrics, 71*(4), 1034-1041. doi:<https://doi.org/10.1111/biom.12349>

Touraine, C., Helmer, C., & Joly, P. (2013). Predictions in an illness-death model. *Statistical Methods in Medical Research, 25*(4), 1452-1470. doi:10.1177/0962280213489234

Williams, C., Lewsey, J. D., Briggs, A. H., & Mackay, D. F. (2016). Cost-effectiveness Analysis in R Using a Multi-state Modeling Survival Analysis Framework: A Tutorial. *Medical Decision Making, 37*(4), 340-352. doi:10.1177/0272989X16651869
